# Bifurcated rib with vertebral defects – A rare anatomical variant: Case report with literature review

**DOI:** 10.1016/j.ijscr.2020.02.010

**Published:** 2020-02-07

**Authors:** Sana Zeeshan, Syeda Namayah Fatima Hussain, Zeeshan Mughal, Shayan Sirat Maheen Anwar, Syed Nadir Naeem

**Affiliations:** a Section of Breast Surgery, Department of Surgery, The Aga Khan University, P.O. Box 3500, Stadium Road, Karachi, 74800, Pakistan; b Liaquat National Hospital and Medical College, Stadium Road, Karachi, 74800, Pakistan; c Department of Neurosurgery, Patel Hospital, ST-18، Block 4 Gulshan-e-Iqbal, Karachi, 75300, Pakistan; d Section of Women’s Imaging, Department of Radiology, The Aga Khan University, P.O. Box 3500, Stadium Road, Karachi, 74800, Pakistan; e Department of Cardiac Surgery, St. George’s University Hospital, Blackshaw Road, Tooting, London, SW17 0QT, United Kingdom

**Keywords:** Bifid ribs, Vertebral defects, Gorlin Syndrome, Bifurcated ribs, Sternum bifidum

## Abstract

**Introduction:**

Bifurcated ribs are a neuroskeletal anomaly which are incidentally discovered on chest X-ray. It is a developmental disorder where sternal end of a rib is split into two, commonly found in males, on the right side and unilaterally not associated with a vertebral defect.

**Presentation of case:**

In this report we discuss a case of a 17-year-old girl with a left bifid rib alongside a segmental defect of the vertebra.

**Discussion:**

Bifurcated ribs usually occur in isolation or with other rib abnormalities associated with vertebral defects. Most cases are asymptomatic or found at post-mortem. Gorlin Syndrome is a condition where both these anatomical defects form a part of its criteria.

**Conclusion:**

With barely any data on significance of bifid rib with vertebral defects and no conclusive exclusion of Gorlin Syndrome, close monitoring coupled with regular follow-ups in such patients is the likely plan of treatment. In addition, genetic testing for susceptible genes should be strongly considered.

## Highlights

• Bifid ribs with vertebral defects and no other clinical manifestation is not a sign of any medical or surgical intervention.
• Such patients should be comprehensively screened by multidisciplinary team to exclude other differentials, especially Gorlin syndrome.
• Regular follow-ups should always be recommended to monitor early onset of nevoid basal cell carcinoma.
• Patient and family education about the possibility of Gorlin syndrome in such situations should be done.

## 1 Introduction

Congenital anomalies of ribs are divided into structural and numerical categories. While numerical abnormalities include an extra or missing rib, structural ones comprise of bifurcated, fused, hypoplastic, forked or bridging ribs [1,2]. Sternum bifidum [3], commonly known as bifid rib, is an anatomic malformation of the anterior chest wall present since birth. Bifurcated ribs always have a forked sternal end which is often, unilateral. This is a rare skeletal abnormality being found in 1.2% of humans making up 20% of congenital rib defects [2,4].

In contrast to other rib malformations, bifid ribs generally occur in absence of vertebral anomalies [5]. Third and fourth ribs are the ones usually involved [6,7].

Generally, the diagnosis is made incidentally, on X-rays or at post-mortem examination. In most cases, it is asymptomatic but previous studies have revealed its presence in genetic syndromes i.e. Gorlin Syndrome, also known as nevoid basal cell carcinoma syndrome, which is caused due to mutation in PTCH1 [6,8,9]. No clinical importance of isolated bifid ribs has been established as yet.

In this report, we describe the case of a 17-year-old girl who presented with a swelling in the left upper chest wall close to breast, which on CT scan was found to have a bifid anterior rib and a fused thoracic vertebra. Owing to the paucity of data about coexistence of bifid ribs and vertebral defects together, this case presents a unique insight in the approach to this clinical condition.

The ethical review committee’s approval was taken for this study. Written informed consent was obtained from the patient’s guardian for publication of this case report and accompanying images. A copy of the written consent is available for review by the Editor-in-Chief of this journal on request. The work has been reported in line with the SCARE criteria [10].

## 2 Presentation of case

A 17-year-old girl, with no prior systemic illnesses, presented to the breast clinic of a secondary hospital with complaint of a lump in her left breast from the last 1 year which was unchanged in size.

Her family history for breast cancer or any other malignancy and congenital spinal syndromes was insignificant. The patient had no history of any trauma in the past.

The patient on inspection showed no visible deformity of face or limbs. Her head circumference was 54 cm (21.5 in.) which was normal for her age and built. On examination, she had a hard palpable swelling at the level of left 2nd and 3rd intercostal space. It was roughly about 2 × 3 cm in size while merging into the adjacent rib. There were no clinically significant findings in her breast or axilla.

**Fig. 1:**
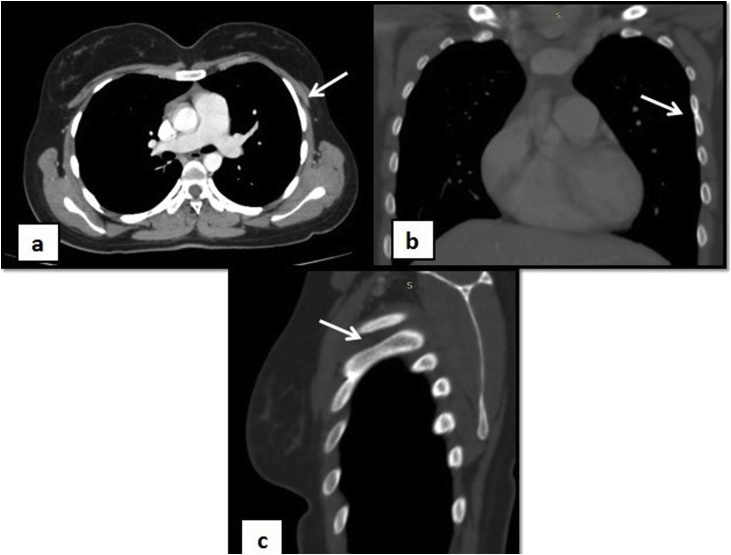
Contrast enhanced Computed tomography (CT) chest shows normal bilateral breast tissue with bifid rib on left.

**Fig. 2:**
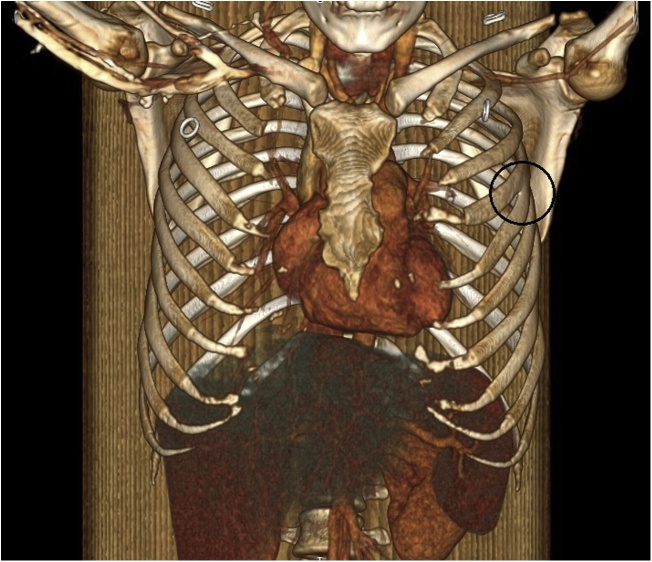
Three-dimensional volume rendering computed tomography (3D-CT) image demonstrates a left 4th bifid rib (encircled).

**Fig. 3:**
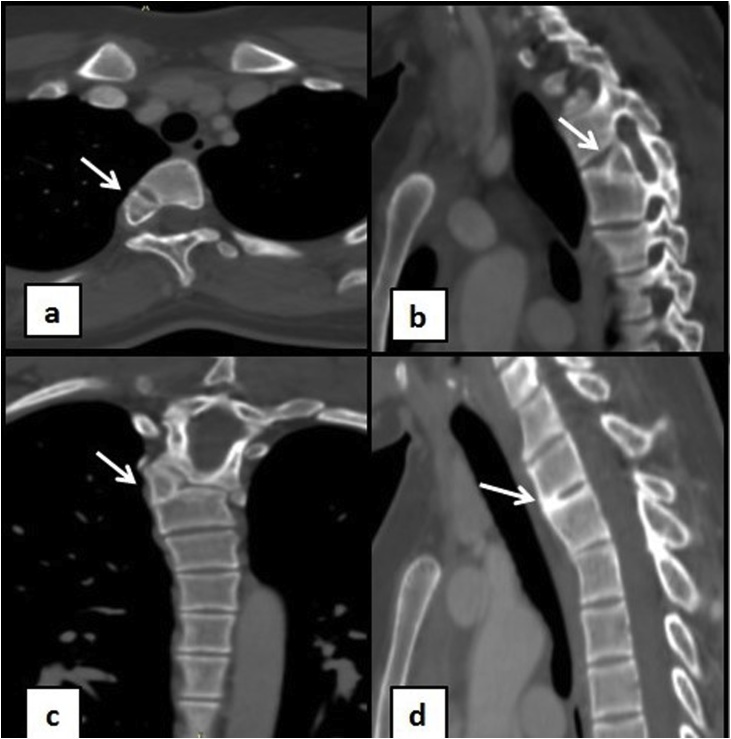
Different CT chest views depicting a 4th hemivertebra. 3a: Axial image, 3b: Sagittal image, 3c: Coronal image showing subtle scoliosis, 3d: Sagittal CT chest image shows a partial block T2 and T3 vertebrae.

Her radiological workup comprised of an ultrasound (U/S) breast followed by Computed Tomography (CT) chest with contrast. The U/S revealed no abnormality in her breasts. However, her CT scan revealed left 4th bifid anterior rib (Figs. 1, 2). A segmentation defect was also identified by the presence of a T4 hemivertebra (Fig. 3a–c) resulting in subtle scoliosis of spine at this level with convexity towards right side and a partially fused T2 and T3 vertebrae anteriorly (Fig. 3d). With the incidental finding of these skeletal abnormalities, her abdominal viscera were also evaluated with CT scan and no abnormalities were identified.

Since the patient was asymptomatic with no other remarkable findings, no treatment was needed. The patient is currently doing well. She merits genetic testing, particularly to analyze PTCH1 gene, to rule out Gorlin syndrome which may predispose her to various cancerous and noncancerous tumors. However, due to unavailability of this test in Pakistan, high cost associated with it if sent abroad and multiple social and cultural issues, the family has declined to pursue this investigation. Hence, regular follow-up has been recommended to ensure no new complications or symptoms occur in the future. This would comprise of yearly skin, breast and dental examinations, orthopentogram every 12–18 months and ovarian ultrasound at 18 years.

## 3 Discussion

Bifurcated ribs usually occur where the sternal end of the rib and costal cartilage meet. It has been postulated that mesodermal abnormalities during embryogenesis could be a cause of this anomaly due to faulty fusion or chondrification. Bifid ribs seem to have a predilection for males as well as the right side of the chest wall [11]. Up to 8.4% frequency found in Samoans displays that ethnicity may play a role in this anatomical defect [8].

Despite little to no information present about the clinical implications of bifid ribs, previous studies support the need for a thorough investigation to rule out Gorlin syndrome [5,12].

Davran et al., in their retrospective review for congenital rib abnormalities in 650 patients, found a 6.76% (44 cases) incidence of bifid rib in the thoracic region with a mean age of 21.1 ± 4.9 years (range: 5–45 years) and a male predilection (82%) [13].

As depicted by the literature search, bifid ribs have mostly been described alone or with other ribs anomalies alongside vertebral defects [5]. Many of the documented cases have been found on routine dissections and bone studies [14,15]. The only scenario with clinical significance where both these defects coexist is Gorlin syndrome [9]. One case with similar findings as ours was reported in a neonatal skeleton postmortem [16]. Gorlin syndrome was considered the main contender of diagnosis for the aforementioned case, largely based on bifid ribs since the other manifestations correlated to no other disease. Bifurcated ribs were established as a part of Gorlin syndrome in 1960 and currently are also a major criterion for diagnosis [8,17]. Vertebral anomalies form one of the minor traits of the syndrome [17].

Gorlin syndrome is documented elaborately in the medical literature. It is an autosomal dominant condition with a prevalence of about 1 in 57,000 [18]. It is characterized by odontogenic keratocysts of the jaw and multiple basal cell carcinomas (BCC) with an increased affinity for developing medulloblastoma in childhood. Development of cardiac fibromas in infants and ovarian fibromas in women necessitate the need for screening in this syndrome [19]. Renal cysts, facial deformities, macrocephaly, palmar pits and congenital skeletal abnormalities are some of its minor manifestations. Our patient had congenital skeletal abnormalities but no other features related to Gorlin syndrome were found. However, adolescence and early adulthood is the most sensitive period with the median age of onset for BCC being 25 years, therefore, our patient requires to remain under regular follow-ups [20].

In this case, the patient was a female of South Asian descent with a bifid rib on the left side. She was asymptomatic with no other characteristics of Gorlin syndrome. Although since she is 17 years of age, there is a likely chance for her to develop tumor or cysts in the future if Gorlin syndrome cannot be ruled out by genetic test.

## 4 Conclusion

This case proves the need to examine all patients presenting with a chest swelling, bifid ribs and segmentation defect with Gorlin syndrome the first on the list of differential diagnosis. Genetic testing would be the definitive testing of exclusion and should be recommended. However, in countries like ours where easy accessibility and cost is a factor, regular checkups should be advised to detect presence of tumors, if any, at the earliest stage ensuring a better prognosis and unimpaired quality of life.

## Declaration of Competing Interest

None.

## Funding

None.

## Ethical approval

Approval as exemption has been taken from Ethical Review Committee of The Aga Khan University with manuscript number: 2019-1413-3584.

## Consent

Written informed consent was obtained from the patient’s guardian for publication of this case report and accompanying images. A copy of the written consent is available for review by the Editor-in-Chief of this journal on request.

## Authors contribution

Dr. Sana Zeeshan - Conception and design, Acquisition of data, Drafting the article, Critical revision of the article, Final approval of the version to be published.

Dr. Syeda Namayah Fatima Hussain - Acquisition of data, Writing the paper, Critical revision of the article, Final approval of the version to be published.

Dr. Zeeshan Mughal - Acquisition of data, Critical revision of the article, Final approval of the version to be published.

Dr. Shayan Sirat Maheen Anwar - Acquisition of data, Critical revision of the article, Final approval of the version to be published.

Dr. Nadir Naeem Syed - Critical revision of the article, Final approval of the version to be published.

## Registration of research studies

None.

## Guarantor

Dr. Sana Zeeshan.

## Provenance and peer review

Not commissioned, externally peer-reviewed.
